# Case report: The first description of a thyroglossal duct cyst in a hen (*Gallus gallus domesticus*)

**DOI:** 10.3389/fvets.2024.1388152

**Published:** 2024-04-22

**Authors:** Romelia Pop, Stephanie Oren, Andrada Negoescu, Cornel Cătoi, Alexandru-Flaviu Tăbăran

**Affiliations:** ^1^Department of Anatomic Pathology, Faculty of Veterinary Medicine, University of Agricultural Sciences and Veterinary Medicine, Cluj-Napoca, Romania; ^2^Departament of Pathology, Kimron Veterinary Institute, Bet Dagan, Israel

**Keywords:** thyroglossal duct cyst, hen, embryological defect, histopathology, first description

## Abstract

Thyroglossal duct cyst represents a congenital anomaly of the cervical region, rarely documented in animals. Although previously reported in dogs, cats, horses, goats, pigs, and calves, never in birds. This report describes a rare case of thyroglossal duct cyst in a hen. A necropsy of a Transylvanian Naked Neck hen carried following diphtheroid mucocutaneous lesions. The necropsy revealed a large, cyst-like structure measuring 0.5 cm at the level of the caudal edge of the left thyroid gland. Histologically, the cystic mass, bordered by 1–2 lines of well-differentiated ciliated cuboidal cells, presented nuclear immunoreactivity for Thyroid transcription factor 1. To the best of the authors’ knowledge, there are no previous records of thyroglossal duct cysts in avians. Moreover, this is the first case describing a thyroglossal duct cyst in a hen.

## 1 Introduction

Thyroglossal duct cyst (TDC), or thyroglossal tract remnant (TTR), is a congenital anomaly of the cervical region that is rarely documented in animals and is caused by incomplete obliteration of the embryonic thyroglossal duct that connects the thyroid gland to the base of the tongue during the first 5 days of embryological life (1, 2).

Although rarely reported in animals, TDCs are mostly described in dogs (3) and cats (4, 5), but they can occur in other species as well as horses (6), goats (7), pigs (8), and calves (9). Interestingly, TDC is one of the most frequent congenital malformations in goats, representing 13% of all congenital changes in a study carried out in Shami goats (7). However, in birds, the only cervical cysts described are branchial cysts, previously described in two Amazon parrots (*Amazona species*) (10), umbrella cockatoo (*Cacatua alba*) (11) and yellow-crested Cockatoo (*Cacatua sulphurea*) (12).

TDC is the most common congenital non-odontogenic cyst of the head and neck in humans, affecting approximately 7% of the population and representing about 75% of the congenital masses of the neck (13, 14), primarily seen in children and young people. Although generally subclinical, TDCs can be associated with local complications such as infection, airway obstruction, and difficulty swallowing. Recurrence might occur in case of incomplete removal of the cyst (15). In dogs and cats, several reports are suggesting a possible malignant transformation of TDC, leading to thyroglossal duct carcinoma (16, 17). The location of the TDC is variable, and it can be found anywhere in the neck region between the base of the tongue and the suprasternal region. According to a study made by Taha in 2022 on 46 patients, the most common sites are infrahyoid (70.2%), suprahyoid (25.5%), and intralingual (4.3%) (15).

This study describes the macroscopic and histological features observed in a rare case of thyroglossal duct cyst in a hen, along with the pathogenesis and the importance of TDCs from the perspective of comparative medicine. To the best of the authors’ knowledge, this is the first report of a thyroglossal duct cyst in avian species.

## 2 Case description

**Figure 1:**
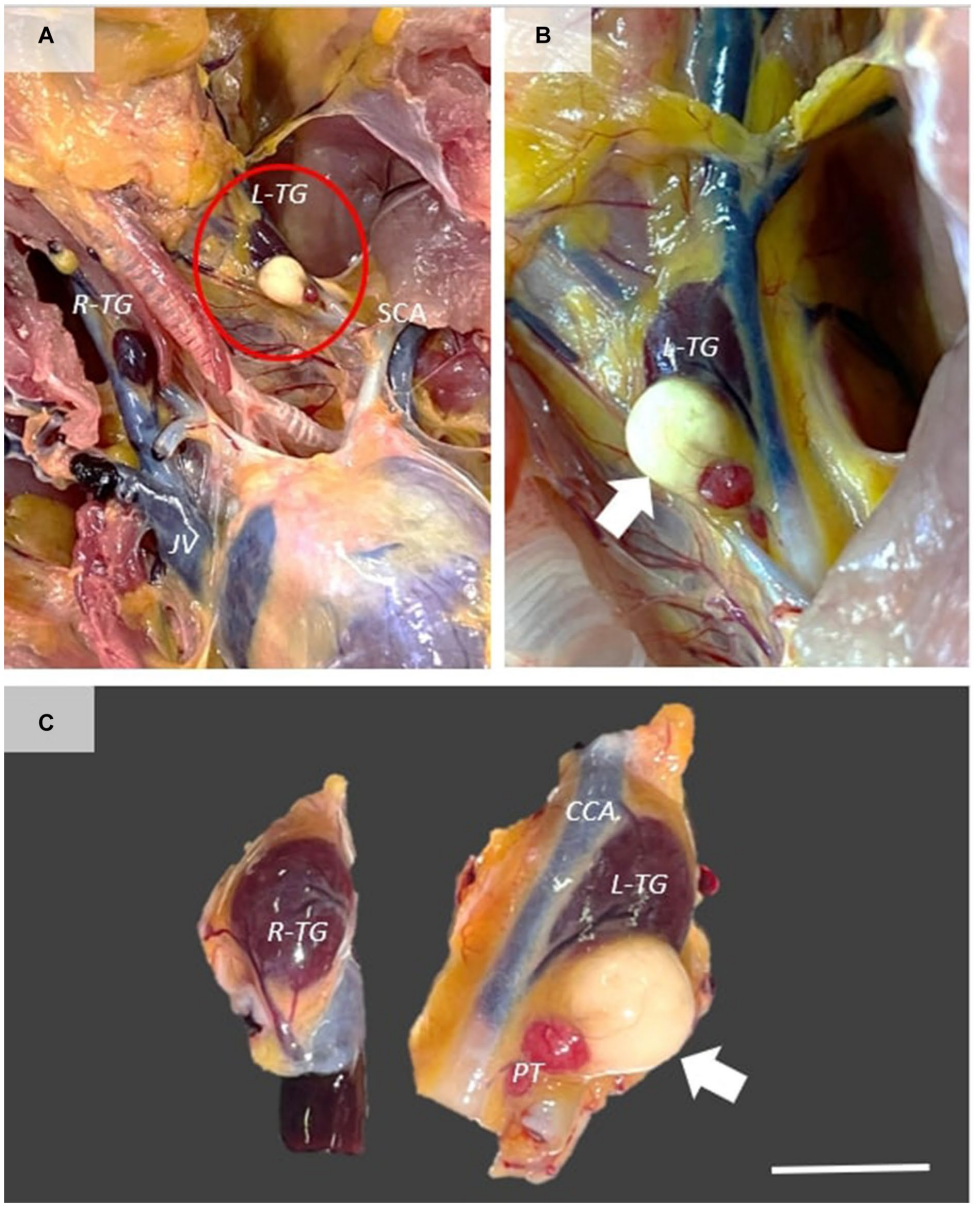
Gross features of thyroglossal duct cyst. **(A)** Ventral view of the cervical and cranial extremity of the thoracoabdominal cavity, showing the presence of a large mass directly attached to the left thyroid gland (white arrow). **(B,C)** The mass is well demarcated, showing moderate compression of the adjacent thyroid gland. Scale bar = 0.5 cm. R-TG, right thyroid gland; L-TG, left thyroid gland; SCA, subclavicular artery; CCA, common carotid artery; PT, parathyroid gland.

A 3-year-old, back-yard Transylvanian Naked Neck hen was submitted for necropsy following a chronic episode of ulcerative and diphtheroid mucocutaneous lesions. The necropsy was carried out at the Pathology Department of the University of Agricultural Sciences and Veterinary Medicine Cluj-Napoca, Romania. The animal presented multifocal nodular and ulcerative cutaneous lesions at the head, neck, and chest level, as well as multifocal-coalescing diphtheroid stomatitis and conjunctivitis. A large, white-gray, well-demarcated, dense, cyst-like structure measuring 0.5 cm was present in the caudal edge of the left thyroid gland. The mass was directly attached to the caudal edge of the thyroid gland and showed moderate compression of the adjacent parenchyma (Figure 1).

**Figure 2:**
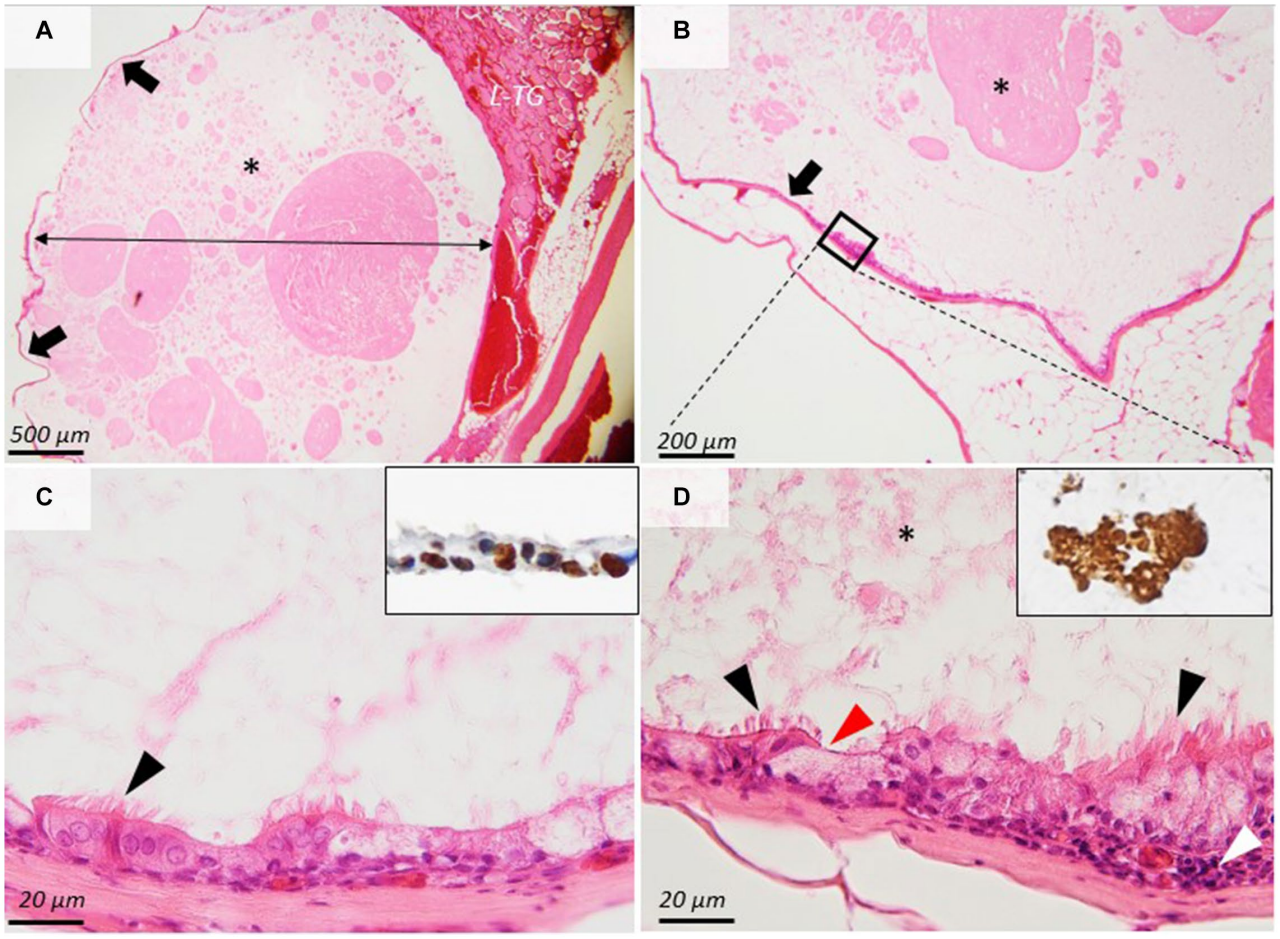
Histopathological images of the thyroglossal duct cyst. **(A,B)** A thin-walled (arrows), well-demarcated cyst (double arrow) contains a large amount of homogeneous, eosinophilic product (asterisks) that is compressing the adjacent thyroid gland (L-TG). **(C,D)** The cyst is bordered by 1–2 lines of ciliated cuboidal cells (arrowheads) and goblet cells that show nuclear immunoreactivity for TTF-1 (**(C)**, inset). The ciliated epithelium is supported by dense fibrous connective tissue. The cyst content is immunopositive for thyroglobulin (**(D)**, inset). H&E stain, ob × 4 **(A)**, × 10 **(B)**, and ×100 **(C,D)**. *A large amount of homogeneous, eosinophilic product.

For histology, the thyroid mass and the adjacent structures were fixed in 10% neutral buffered formalin (NBF) and embedded in paraffin following the routine processing protocol. Two-micrometer histological sections were stained using hematoxylin and eosin (H&E). The histological samples were further examined by immunohistochemistry using a Leica Bond-Max automated immunostainer (Leica Microsystems) for the tissular expression for TTF1 (Mouse Monoclonal Antibody, SPT24 clone, Leica Biosystems, No. PA0364, 1;200) (18) and Thyroglobulin (Mouse Monoclonal Antibody Thyroglobulin, 1D4 clone, Leica Biosystems No. PA0025, used dilution 1:200). The immunohistochemistry protocol using a polymer-based detection system (Leica Biosystems, nr. DS9800) having as a chromogen 3, 3-Diaminobenzidine (DAB) was carried out by the protocols provided by the auto-immunostainer producer. The histopathological exam showed a thin-walled, well-demarcated singular cyst that contained a large amount of homogeneous, pale-eosinophilic product immunopositive for thyroglobulin. The cyst was bordered by 1–2 lines of ciliated cuboidal to columnar cells admixed with fewer goblet cells. Mitotic figures were absent. The cells were supported by a thin layer of dense-fibrous connective tissue containing few blood vessels, separating the cyst from the adjacent thyroid parenchyma and adipose tissue. The epithelial cells showed a nuclear immunoreactivity for TTF-1, demonstrating the thyroidal origin (Figure 2). According to the histological and immunohistochemical findings, the diagnosis of Thyroglossal duct cyst was performed.

## 3 Discussion

The main differential diagnoses for cyst-like structures in birds’ neck areas include branchial cysts, bronchogenic cysts, ultimobranchial body cysts, thymic epithelial cysts, benign or malignant tumors, goiter, and salivary gland cysts, but the anatomic site, histologic lining, and identification of thyroid tissue help with this separation.

Branchial cysts arising from the branchial cleft are lined by polymorphic neoplastic squamous epithelium with prominent mitotic activity, admixed with multiple lymphoid aggregates, forming fine projections into the cystic cavity. Branchial cysts that originate from branchial pouches are multicystic, lined by tall columnar epithelium, and produce a mucoid material that distends the cystic cavity (10). In TDC, the cystic wall is lined by a thin layer of well-differentiated ciliated epithelium. Bronchogenic cysts are lined by columnar, ciliated, pseudostratified epithelium associated with prominent mesenchymal elements such as blood vessels admixed with hyaline cartilage, smooth muscles, and elastic fibers (19), the last elements being absent in TDC. The ultimobranchial body cyst is usually lined by cuboidal epithelium, occasionally with the presence of short, irregular microvilli, while the TDC is lined by ciliated columnar cells admixed with few goblet cells. The TTF1 positivity for TDC represents one of the key elements in diagnostics, while the possible expression of TTF in the utimobranchial body cyst is poorly documented (20). Two types of epithelial cystic structures have been described in birds’ thymic medulla: intracellular cysts formed in a single epithelial cell (also described in mammals) and intercellular cysts formed by two or more epithelial cells (observed only in birds). The thymic epithelial cysts are small (up to 4 μm) (21), lack basal membrane or a typical cystic structure, and are surrounded by lymphocytic aggregates within the thymic parenchyma. Various types of tumors, both benign and malignant, can develop in the cervical area of birds, including thyroid adenocarcinoma (22), lipomas (23), and fibrosarcomas (24). Goiter (thyroid follicular hyperplasia) with an enlargement of the thyroid gland can develop in birds. In the thyroid follicular hyperplasia (goiter), the thyroid follicles are diffusely enlarged and contain homogenous eosinophilic colloid but are lined by flat to low cuboidal follicular epithelial cells (25) and not ciliated epithelia as in the TDC. Salivary gland cysts (mucoceles) in birds were previously described in a spectacled owl (*Pusilatrix perspicillata*) (26) and consist of multilocular cysts lined by cuboidal epithelia containing mucus-rich fluid admixed with foamy macrophages and fewer heterophils.

The thyroglossal duct, also known as the thyroglossal tract, is an embryonic structure that plays a role in the thyroid gland’s development. The thyroid gland is formed in the early embryonic development, initially situated at the base of the tongue and later descends downward to its final position in the neck. The thyroglossal duct is a narrow tube-like structure connecting the developing thyroid gland to the tongue. Usually, as the thyroid gland descends, it gradually disappears. However, the thyroglossal duct may occasionally persist or partially close off, resulting in a thyroglossal duct cyst or fistula (27).

In avian species, including chickens, the embryonic development of the thyroid gland manifests initially as a ventral out-pocketing of the pharynx discernible by day 2 of incubation. The primordial thyroid gland separates from the growing digestive system on day 4 of incubation and remains connected by the thyroglossal duct, a tiny, two-cell layer structure. By day 5 of incubation, the embryological development of the thyroglossal duct, which eventually regresses to a tiny cell isthmus between the two lobes of the adult gland, is complete. At the same time, the thyroid gland develops a bilobed structure and moves to its final location. Along this pathway, functional thyroid tissue may remain ectopically (19, 28, 29). The tissue’s vesicles initially emerge after 9 days of incubation, characterized by cuboidal epithelial cells within them. Subsequently, there is a growth in glandular tissue, along with an increase in both the quantity and size of the vesicles. By the 15th day of incubation, the glands closely resemble those of adult animals, with the epithelial cells within the vesicles becoming mostly flattened (30). A cyst in the thyroglossal duct will occur if this tract is not completely obliterated and the epithelial cells fail to remain quiescent. Once acquired, the secretory activity will continue, resulting in the development of the thyroglossal duct cyst (14, 31).

Thyroglossal duct cyst is reported sporadically in veterinary medicine. This study describes the gross and histopathological features of a rare case of thyroglossal duct cyst in a hen. To the best of the authors’ knowledge, this is the first report of a thyroglossal duct cyst in birds.

## Data availability statement

The original contributions presented in the study are included in the article/supplementary material, further inquiries can be directed to the corresponding author.

## Ethics statement

Ethical approval was not required for the studies involving animals in accordance with the local legislation and institutional requirements because this is a case presentation performed on a dead animal submitted by the owner for a necropsy diagnostic. A formal written consent from the owner allowing teaching and research activity on the sample was obtained at the moment of sample submission. The university ethics committee was consulted, and they responded that formal approval on their part was not required. Written informed consent was obtained from the owners for the participation of their animals in this study.

## Author contributions

RP: Conceptualization, Data curation, Writing – original draft. SO: Writing – review & editing. AN: Investigation, Writing – review & editing. CC: Writing – review & editing. A-FT: Validation, Writing – original draft, Writing – review & editing.
